# A180 CLINICAL OUTCOMES OF COVID-19 AND IMPACT ON DISEASE COURSE IN PATIENTS WITH INFLAMMATORY BOWEL DISEASE

**DOI:** 10.1093/jcag/gwab049.179

**Published:** 2022-02-21

**Authors:** P Wetwittayakhlang, F Albader, P Golovics, G Drügg Hahn, T Bessissow, A Bitton, W Afif, G Wild, P L Lakatos

**Affiliations:** 1 Gastroenterology, McGill University Health Centre, Montreal, QC, Canada; 2 Gastroenterology, McGill University, Montreal, QC, Canada; 3 IBD Centre, McGill University Health Center, Montreal, QC, Canada

## Abstract

**Background:**

The impact of COVID-19 has been of great concern in patients with IBD worldwide, including an increased risk of severe outcomes and/or flare of IBD.

**Aims:**

This study aims to evaluate prevalence, outcomes, the impact of COVID-19 in patients with IBD, and risk factors associated with severe COVID-19 or flare of IBD.

**Methods:**

A consecutive cohort of IBD patients diagnosed with COVID was obtained between March 2020 - April 2021.

**Results:**

A total of 3,516 IBD cohort patients were included. 82 patients (2.3%) were diagnosed with COVID infection (median age 39.0, 77% with Crohn’s disease). The prevalence of COVID-19 in IBD was significantly lower compared to the general population in Canada and Quebec (3.5% vs. 4.3%, p<0.001). Severe COVID occurred in 6 patients (7.3%); 2 patients (2.4%) died. A flare of IBD post-COVID infection was reported in 8 patients (9.8%) within 3 months. Age ≥55 years (OR 11.1, 95%CI:1.8–68.0), systemic corticosteroid use (OR:4.6, 95%CI:0.7–30.1), active IBD (OR:3.8, 95%CI:0.7–20.8) and comorbidity (OR:4.9, 95%CI:0.8–28.6) were associated with severe COVID. After initial infection, 61% received vaccinations.

**Conclusions:**

The prevalence of COVID-19 among patients with IBD was lower than the general population. Severe COVID and flare of IBD were relatively rare. Older age, comorbidities, active IBD, and corticosteroid, but not biological therapy were associated with severe COVID.

Outcome of COVID-19 in IBD patients, disease course of IBD, and vaccination after COVID infection

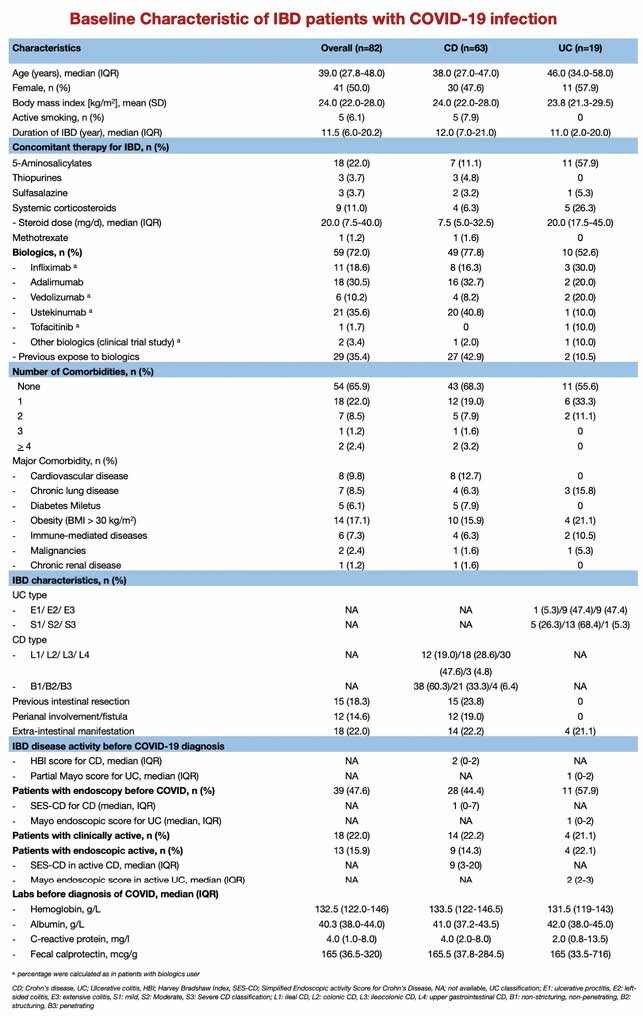

**Funding Agencies:**

None

**Table:** 

COVID-19 outcomes	Number of patients (%)a
Mild COVID-19 symptoms	76 (92.7)
Hospitalization due to COVID-19	6 (7.3)
Duration of hospitalization (day), median (IQR)	10.5 (3.75–29.0)
Recovery day after admission, median (IQR)	12.5 (7.0–25.5)
COVID-19 pneumonia	5 (6.1)
Required mechanical ventilators	2 (2.4)
Required intensive care admission	2 (2.4)
Dead	2 (2.4)
C-reactive protein post-COVID (mg/l), median (IQR)	4 (2.0–12.8)
Fecal calprotectin post-COVID (mcg/g), median (IQR)	197 (57.5 – 653.5)
Flare of IBD disease after COVID-19 infection	8 (9.8)
- Duration after COVID (weeks), median (IQR)	9.5 (7.2–12.0)
- C-reactive protein during flare (mg/L), median (IQR)	8.0 (3.0–42.0)
- Fecal calprotectin during flare (mcg/g), median (IQR)	1,088.5 (562- 2,025)
Treatment care outcomes	
- Loss of follow-up	15 (18.3)
- Disruption of biologics	30 (36.6)
- Duration of holding biologics (week), median (IQR)	3.0 (2.0–5.0)
- Number of patients had delay endoscopy	7 (8.5)
COVID-19 Vaccination	
- 1 dose vaccine	39 (47.6)
- 2 doses vaccine	11 (13.4)

aData are presented as number (%) unless indicated otherwise. CD; Crohn’s disease, UC; Ulcerative colitis, NA; not available, IQR; interquartile range,

